# Temperature as a key parameter for graphene sono-exfoliation in water

**DOI:** 10.1016/j.ultsonch.2022.106187

**Published:** 2022-09-30

**Authors:** Amanpreet Kaur, Justin A. Morton, Anastasia V. Tyurnina, Abhinav Priyadarshi, Adam Holland, Jiawei Mi, Kyriakos Porfyrakis, Dmitry G. Eskin, Iakovos Tzanakis

**Affiliations:** a School of Engineering, Computing and Mathematics, Oxford Brookes University, College Cl, Wheatley, Oxford OX33 1HX, UK; b Brunel Centre for Advanced Solidification Technology, Brunel University London, Kingston Lane, UB8 3PH, UK; c Kyoto Cl, Moulton Park Industrial Estate, Moulton Park, Northampton NN3 6FL, UK; d Department of Engineering, University of Hull, Cottingham Rd, Hull HU6 7RX, UK; e Faculty of Engineering and Science, University of Greenwich, Central Avenue, Chatham Maritime, Kent ME4 4TB, UK; f Department of Materials, University of Oxford, Parks Rd, Oxford OX1 3PH, UK

**Keywords:** Ultrasonic processing, Cavitation bubbles, Shock waves, Graphene, Exfoliation, Eco-friendly, Water

## Abstract

Graphene dispersions in water are highly desirable for a range of applications such as biomedicines, separation membranes, coatings, inkjet printing and more. Recent novel research has been focussed on developing a green approach for scalable production of graphene. However, one important parameter, which is often neglected is the bulk temperature of the processing liquid. This paper follows our earlier work where optimal sono-exfoliation parameters of graphite in aqueous solutions were determined based on the measured acoustic pressure fields at various temperatures and input powers. Here, we take the next step forward and demonstrate using systematic characterisation techniques and acoustic pressure measurements that sonication-assisted liquid phase exfoliation (LPE) of graphite powder can indeed produce high quality few layer graphene flakes in pure water at a specific temperature, i.e. 40 °C, and at an optimised input generator power of 50%, within 2-h of processing. UV–vis analysis also revealed that the exfoliation, stability and uniformity of dispersions were improved with increasing temperature. We further confirmed the successful exfoliation of graphene sheets with minimal level of defects in the optimized sample with the help of Raman microscopy and transmission electron microscopy. This study demonstrated that understanding and controlling processing temperature is one of the key parameters for graphene exfoliation in water which offers a potential pathway for its large-scale production.

## Highlights

• Control of temperature is the key for successful graphene exfoliation in water.
• Increase in solution temperature reduces the intensity of the shockwave (SW) peak.
• SWs wavelength was kept at 430–460 µm not being affected by the temperature.
• Optimum balance of SWs and bubble clouds at 40 °C produced the efficient exfoliation.
• High quality few-layer graphene with an area of ∼0.6 μm^2^ produced in 2 h.

## 1 Introduction

Among the most reliable exfoliation techniques, ultrasound assisted liquid phase exfoliation (ULPE) is considered as a facile, cost-effective and scalable process to produce variety of two dimensional (2D) layered materials in a benign fashion [1]. The resulting liquid-suspended 2D nanosheets can be further exploited for numerous applications such as gas sensors [2], [3], cytotoxicity of cells, drug delivery [4] and water filtration [5], to name but a few. Owing to the versatility of ULPE, it has become a technique of great interest amongst the graphene research community. On the other hand, there are a plethora of existing routes for producing graphene which mainly include micro-mechanical exfoliation [6], chemical vapor deposition (CVD) growth of graphene [7] and chemical oxidation of graphite [8]. Simultaneously, it is also apparent that the above-mentioned three methodologies suffer from scalability, cost-effectiveness and quality issues. As a prerequisite of understanding fundamental properties of graphene and recognizing its real world applications, methods for preparing graphene should be currently focussed on ensuring eco-friendly, economic and high-throughput 2D materials.

Earlier, promising results via sonication-assisted exfoliation method were obtained using toxic organic solvents such as dimethylformamide (DMF), *N*-methyl-2-pyrrolidone (NMP) and tetrahydrofuran (THF) etc. as the intercalating mediums [9]. However, the use of aforesaid toxic organic solvents might be detrimental for both ecological and biological applications. Moreover, post-exfoliation removal of the organic solvents during purification requires large quantities of solvents which are usually expensive and need special care while handling [10]. Therefore, in view of the toxicity of solvents finding a green solvent for ULPE is a pressing demand to bring graphene closer to its real world applications.

In this scenario, water being eco-friendly, easy to handle, cost-effective and an abundant dispersion medium is considered to be a frontliner especially after being recently proved to be hydrophilic to graphene [11]. The aqueous based ULPE technique bypasses the usage of toxic agents like alkali metals, organic solvents and additional capping agents that degrade the original conjugated structure of graphene and, therefore, make it adverse for device applications [9], [10]. Although, exfoliating graphene in pure water is a challenging task, by optimising and controlling the input power and bulk temperature of water during ultrasonic processing, we can alleviate this problem to a great extent and produce flakes of high quality. In particular, our group recently developed a technique that generated high quality few layer graphene (FLG) flakes in a range of 3–5 layers (Ls) using a dual frequency approach in pure water [12], [13] under controlled temperature. However, the particular temperature of 40 °C has been empirically chosen based on the existing literature without a solid scientific rationale behind this choice, and this what we aim to address in this work.

To the best of our knowledge, the effects of temperature on the graphene exfoliation in deionized water (DIW) have not been reported yet, despite a few earlier attempts in the literature. For example, Kim *et al.* [14] investigated the effect of temperature (30 °C and 60 °C) for the exfoliation of two-dimensional (2D) nanoplatelets (graphene, h-BN, MoS_2_, WS_2_, and MoSe_2_) in water using a 40 kHz bath-sonication for 60 h. Another study featured the effect of temperature on the graphene exfoliation in the presence of sodium cholate with a shear mixing technique [15]. Furthermore, S Kumar *et al.* [16] highlighted the role of temperature-controlled exfoliation on the properties of graphene oxide sheets in water. Moreover, current reports on direct exfoliation of graphite mainly focus on solvent selection [17], sonication duration [18], input powers [19], centrifugation rate [20] and choice of starting material [21]. Hence, these studies do not take into account the effect of bulk solution temperature on the exfoliation.

For this reason, this article is specifically aimed at finding the one-step facile ULPE route for producing graphene in DIW at an optimum solution temperature based on the role of cavitation dynamics and following our previous work in [22], which opens the way for its exploitation in a wide spectrum of applications where graphene in pure water is the first choice.

## 2 Materials and methods

**Fig. 1:**
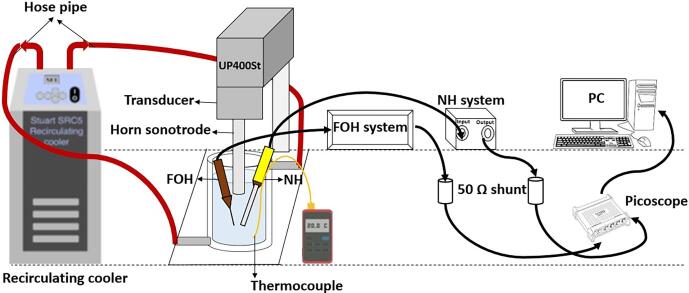
The schematic illustration for performing ULPE of graphene coupled with acoustic detection equipments.

A double walled borosilicate glass beaker (Cole Parmer, 250 ml, 50 mm-diameter) filled with 150 ml ultra-pure deionized water (Hexeal Chemicals, UK) was integrated to a recirculating cooler (Cole Parmer Stuart SRC5) through hose pipes allowing for temperature control. Fig. 1 shows the schematic diagram of our low-frequency experimental setup used for performing ULPE of graphene. The series of ULPE experiments were performed using a Heilscher UP400St piezo-electric transducer (a titanium sonotrode tip diameter of 22 mm, operating at a frequency of 24 kHz) in DIW at different temperatures 10 ± 1 °C, 20 ± 1 °C, 40 ± 1 °C and 60 ± 1 °C (verified with an RS 52 digital thermometer) for 50 % (peak-to-peak amplitude, 23 μm) based on our previous work [22] and 60 % (for close comparison) input generator powers (peak-to-peak amplitude, 27 μm). The calculated values of acoustic energy and sonication energy for different temperatures are listed in Table 1. We considered acoustic energy to be a more relevant quantification parameter for these particular experiments, where the temperature of the solution is controlled by the recirculating chiller. It is also a common practice in sono-studies to use acoustic input energy or probe displacement (peak-to-peak amplitude) facilitating direct comparison with other works in the field [22], [46], [47]. The probe tip was immersed 10 mm below the liquid surface for all the experiments. The initial concentration of graphite powder (GP) (Alfa Aesar 300 mesh, particle size of maximum 56 μm was used as received without any further modification) was 0.4 g/l (60 mg in 150 ml of water). Acoustic energy is transformed to heat, increasing the temperature of the solution, which can be controlled by the cooling contour to maintain the set temperature. Initially, the temperature was sustained constant with both sonication (without adding GP) and the chiller on. When the desired temperature was achieved, pre-weighed GP was added to the DIW and stirred to disperse homogeneously with continous sonication for 2 h. Immediately after 2 h of ULPE, dark graphene poly-dispersions of approximately 10 ml were pipetted and centrifuged at 1500 *g* RCF (relative centrifugal force measured in the units of gravity, i.e., here is the g-force in m/s^2^) for 30 min using SciSpin One Benchtop centrifuge to sediment un-exfoliated graphite particles/thick flakes to obtain supernatants. Subsequently, UV–vis absorption spectra of as-obtained fresh supernatants were recorded in the wavelength range of 200–800 nm with a Cary-60 spectrophotometer (Agilent Technologies) using quartz cuvettes (volume 3.5 ml, an optical path length of 10 mm, Agilent Technologies). Dual-beam mode and baseline correction were used throughout the measurements to scan the samples. It is to be noted that experiments were repeated three times for each combination of parameters to validate the consistency in results. After getting reasonable findings from the preliminary UV–vis measurements, the examined supernatants were drop-cast onto a cleaned silicon substrate (Diameter 3″, Orientation 〈1 0 0〉 from Pi-Kem, UK) and subsequently dried in a vaccum oven prior to Raman investigations. Further, micro-Raman analyses of the drop-cast samples were performed using a Horiba LabRAM HR Evolution confocal Raman spectrometer with 532 nm excitation. Data collection was performed in the range from 1200 to 3100 cm^−1^ using a 100 × objective with an average of 10 s acquisitions and used automated cosmic ray removal. Simultaneouly, 2–3 drops were put onto holey carbon coated copper grid (300 mesh, purchased from Agar Scientific, UK) placed on a filter paper to wick away excess solvent and was dried completely for transmission electron microscopy (TEM) investigations. TEM analyses were performed to interrogate the individual flakes using a JEOL 2100F Field Emission Gun operating at 200 kV.

**Table 1:** The enlisted values of acoustic intensity (W/m^2^) and sonication energy (kJ/ml) in DIW at different temperatures.

Temperature (^o^C)	P_liquid_ (W)	P_liquid_- P_air_* (W)	Acoustic# Intensity (W/m^2^) × 10^4^	Sonication^@^ energy, E (kJ/ml)
50 % power				
10	95	78	20.58	3.74
20	94	77	20.31	3.69
40	90	73	19.26	3.50
60	80	63	16.62	3.02
60 % power				
10	114	94	24.80	4.51
20	110	90	23.74	4.32
40	108	88	23.21	4.22
60	95	75	19.78	3.60

*P_air_ = 17 W (50 %, peak to peak amplitude, 23 μm); *P_air_ = 20 W (60 %, peak to peak amplitude, 27 μm).

^#^Acoustic intensity= $\frac{\;\;\;\;\;\;\;P_{liquid}-P_{air}\;\left(W\right)}{\mathrm{Area}\;\;of\;\;sonotrode\;\left(m^{2}\right)}$; @ Sonication energy =.$\frac{\;\;\;\;\;\;\;\left(P_{liquid}-P_{air}\;\left(W\right)\right)\times sonication\;\;time\;\left(s\right)}{\mathrm{Volume}\;\;of\;\;solution(ml)}$

Area of sonotrode (22 mm diameter) = 3.79 × 10^-4^ m^2^; volume of liquid = 150 ml; Sonication time = 2 h (7200 s).

The cavitation intensity in the solution, under the experimental parameters was monitored using two advanced calibrated acoustic sensors. A 10 μm diameter fibre-optic hydrophone (FOH, Precision Acoustics ltd) calibrated between 300 kHz and 30 MHz and a 4 mm diameter needle hydrophone (NH, Precision Acoustics ltd) calibrated between 8 and 400 kHz were positioned ∼2.5 cm underneath the sonotrode as shown in Fig. 1. Using these two sensors, acoustic emissions were captured from a broad range of frequencies associated with cavitating and collapsing bubbles and corresponding shock wave (SW) emissions [23]. Acoustic signals captured by both sensors were converted into raw voltage signal and recorded by an external digital oscilloscope device (PicoScope 3000 series). Real-time signal monitoring of the cavitation activities captured 60 signals within a 2 ms period, resulting in a total of 120 ms. The entire analysis of the experimental acoustic data was carried out via an in-house MATLAB code based on the deconvolution process as described in [24], [25].

## 3 Results and discussion

### 3.1 UV–vis spectral analysis

**Fig. 2:**
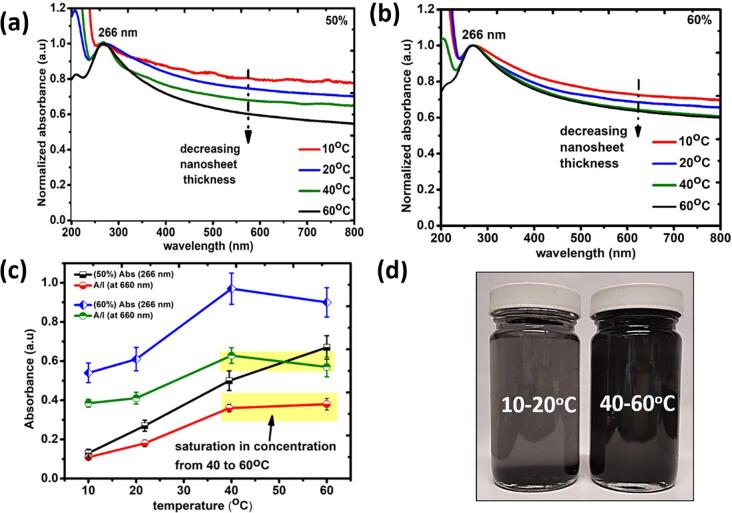
(a), (b) normalized UV–vis absorption spectra obtained for graphene supernatants after ULPE at different temperatures for 50 % and 60 % input powers respectively; (c) plot featuring the trend of Abs (266 nm) and A/ℓ (660 nm) (Y-axis) as a function of processing temperature (X-axis) for both 50 % and 60 % input powers; (d) post-2 h sonication (without centrifugation) obtained slurries showing poor dispersibility at 10–20 °C (greyish transparent) in comparison to 40–60 °C (black).

Fig. 2 (a) and (b) present the normalized graphs derived from recorded UV–vis absorption spectra of obtained supernatants after ULPE processing at 10 °C, 20 °C, 40 °C and 60 °C for 50 % and 60 % input generator powers respectively. In the first instance, the peaks centred at ∼266 nm, characterstics absorption peaks of graphene ascribed to π-π* [26] are found in all the processed samples. From the normalized graphs given in Fig. 2(a) and (b), discernible variations in the slopes of the curves provide the information related to thickness of nanosheets [26], which is indicated with dashed arrow. Interestingly, we observed that as the solution temperature increased, there was a drop in the thickness of exfoliated sheets (pronounced thinning effect of the sheets). However, saturation in the shape of slopes at 40 °C and 60 °C for the samples processed at 60 % input power was also noticed in Fig. 2(b). For further understanding, Fig. 2 (c) highlights the plot between the maximum absorption peak intensity (∼266 nm) and the qualitative concentration A/ℓ (at 660 nm) (in accordance with Lambert-Beer‘s law, A/ℓ (at 660 nm) = αC where A is measured absorbance, ℓ is optical path length, α is extinction coefficient (1390–6600 ml. mg^−1^.m^−1^ [20], [26]) and C is concentration of suspension [20]) as a function of temperature (along X-axis). From Fig. 2 (c), for the 50 % input power, the absorption intensity of graphene related peak (Abs (266 nm)) increased with temperature and this intensity dropped after 40 °C for 60 % input power. Additionally, the concentration tends to saturate after 40 °C for both the 50 % and 60 % input powers which is compelling evidence for selecting a comparatively lower temperature, i.e., 40 °C as opposed to 60 °C. In addition, 40 °C is easier to handle and maintain specifically for scale-up processes, otherwise using a heating element may be required for larger volumes. Moreover, poor dispersibility of GP in water at 10 °C and 20 °C was observed post-sonication in comparison to 40 °C and 60 °C which can be seen in Fig. 2 (d).

**Fig. 3:**
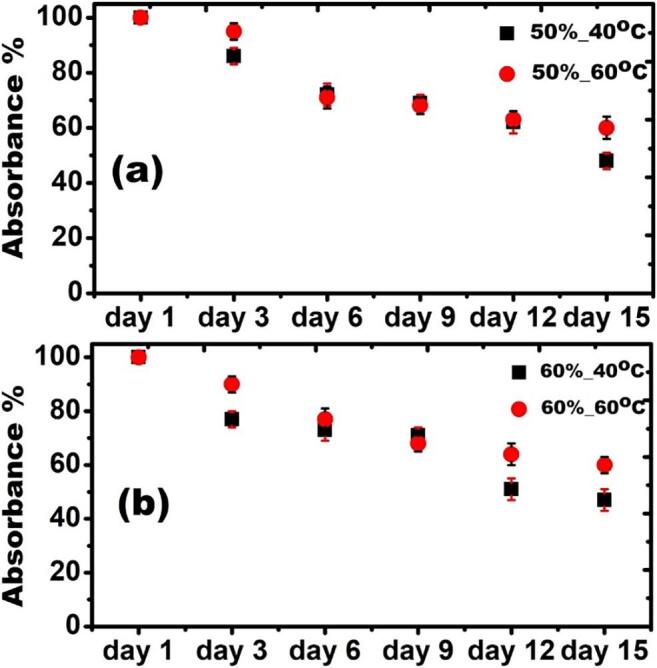
Sedimentation plots of graphene suspension in DIW over 15 days at room temperature; (a) 50 %–40 °C, 50 %–60 °C and (b) 60 %–40 °C, 60 %–60 °C, respectively.

Based on the UV–vis results, we further investigated the stability of the slurries (the amount of retained graphene (A/ℓ (at 660 nm)) after a certain period of time, estimated from Lambert-Beer’s law [20], [27]) of samples of interest, kept at room temperature conditions. Fig. 3 demonstrates the stability investigations of the samples prepared under different input power-temperature conditions i.e., 50 %–40 °C, 50 %–60 °C, 60 %–40 °C and 60 %–60 °C. From the plots, we noticed the trivial differences of sedimentation rate between 50 % and 40 °C, 50 %–60 °C (Fig. 3(a)) and 60 %–40 °C, 60 %–60 °C (Fig. 3(b)) samples up to 9 days. Afterwards, we observed ∼31 % more stability in the samples processed at 60 °C for both the powers. We understand the improved stability of graphene flakes exfoliated in 60 °C samples by assuming the large population of small sized flakes produced in them. In this context, Yi *et al.* [28] correlated the stability of graphene in water with the corresponding flake sizes and proposed that if the flake sizes are significantly reduced, their amount of edge carbon atoms (or dangling atoms located at edges) increases, being reactive in nature, they tend to form bonds with oxygen from the surrounding water molecules, which helps them to remain suspended in a liquid medium under room temperature. Besides, Kim *et al.* [14] also observed the enhanced stability of graphene flakes, which were exfoliated at 60 °C in comparison to 30 °C. Kuziel *et al.* [29] performed density functional theory (DFT), molecular dynamics (MD), Monte Carlo (MC) calculations on size-dependent amphiphilicity of graphene flakes in which small-sized graphene flakes with high edge-to-surface area ratio are proved to be hydrophilic, and these hydrophilic edge sites decrease with increasing lateral size of the flake. From their studies, DFT calculations also revealed that graphene flakes possess two distinctive regions: hydrophobic basal plane surface composed of sp^2^ carbon atoms and hydrophilic edges with dangling bonds. Therefore, oxygen atoms from water molecules preferentially direct toward the hydrophilic edge sites of the flake, which facilitate the stability of flakes in water. Even though, there are several other reported factors such as matching of surface energies between graphene and solvent [30], pH of solvent [31], addition of surfactants [32], non-covalent modification with graphene oxide [33] etc. that explains the stability mechanisms of graphene in a liquid medium, but none of these factors seems conducive to our observations. However, small sized flakes with lateral sizes of several 10 to 100 nm suspended in a liquid have their own merits as they are highly demanding in inkjet printing applications [28].

### 3.2 Raman spectral analysis

**Fig. 4:**
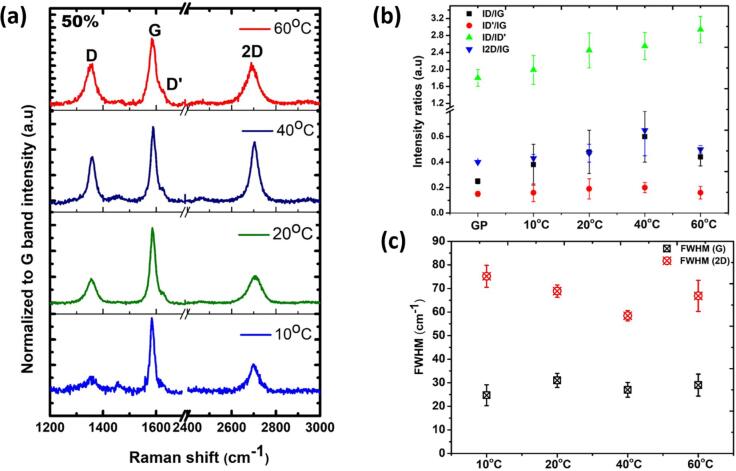
(a) Representative Raman spectra of observed flakes found in each sample processed at 50% input power featuring D, G, D’ and 2D peaks, spectra are normalized to the G band intensity; (b) Averaged intensity ratios of peaks, I_D_/I_G_ (black squares), I_D’_/I_G_ (red circles), I_D_/I_D’_ (green triangles) and I_2D_/I_G_ (blue triangles) of registered flakes in each sample. The data for original GP is also provided alongside for reference; (c) Plot of the FWHM of G and 2D band as a function of processing temperature.

Raman spectroscopy is an effective tool for analyzing the defects and thickness of graphene sheets [34]. Based on previous UV–vis section, there was no significant difference in the results obtained with either of the input powers (50 % and 60 %) as per Fig. 2 and thus for Raman studies, we only considered the samples processed with 50 % input power as a most energy efficient approach. Fig. 4(a) demonstrates the representative Raman spectra of investigated graphene flakes obtained after ULPE at different temperatures 10 °C, 20 °C, 40 °C and 60 °C for 50 % input power. All the spectra are linear baseline subtracted and normalized to G-band intensity. The prominent features of graphene i.e. D, G, D’ and 2D bands positioned at 1350, 1580, 1620 and 2700 cm^−1^, respectively had been registered in each case. Both D and D’ bands are, attributed to the existence of defects such as edges, presence of functional groups and structural disorders [35]. The G band governs the in-plane vibrations of sp^2^ bonded carbon atoms [34], [35]. Variations in line-shape, intensity, position and Full Width Half Maximum (FWHM) of a significant 2D band, the second-order two phonon process of the D band, reveal information on the number of graphene layers and thinning effect [36], [37]. From Fig. 4(a), we can see enhancement in the intensity of D and 2D bands with temperature, which suggests the evolution of structural defects as a result of thinning of GP or, in other words, exfoliation progressing with temperature. For better understanding, we noted the intensity ratios of D, D’ and 2D bands with G band for each recorded spectrum whose average values are provided in Fig. 4(b). From the estimated values given in Fig. 4(b), it has been noticed that defect ratio (*I_D_/I_G_*) (indicated by black squares) values increased up to ULPE at 40 °C followed by its downfall. We understand this observation is typical for solvent-exfoliated graphene flakes, the D band actuates due to the presence of edge sites with more active dangling bonds, produced with the cutting down of lateral size of flakes [37]. There could be a possibility that small sized flakes are formed abundantly in the 60 °C sample, owing to their high surface energy, Van der Waals forces of attraction between them are relatively higher [38], therefore, their degree of agglomeration is expected to be higher after being drop-cast on a substrate, which Raman scans registered an apparently thick material.

To put it in another way, the *I_D_/I_G_* ratio is inversally proportional to length of graphene flakes in accordance with equation (1) [39].

(1) $$\frac{I_{D}}{I_{G}}=\frac{0.26}{<L>}$$

where *I_D_/I_G_* is the defect ratio and <L> is the mean lateral flake size.

The consistent increase in defect ratios until 40 °C advocates the slicing of graphite crystallites which manifests the progress of exfoliation with temperature. Analogues to defect ratios, the increase in *I_D’_/I_G_* ratios (indicated by red circles) with temperature validates that edge defects are growing with temperature while the decline at 60 °C indicates closing of edge sites. Therefore, this disparate behaviour at 60 °C can be explained on the basis of re-stacking of scissored graphene flakes through reactive edge sites with the neighbouring flakes [40]. We further evaluated *I_D_/I_D’_* ratios (indicated by green triangles) for the registered flakes and they were found to be less than 3.5 for each temperature, which indicates the formation of edge defects in accordance to Eckmann *et al.* studies [41]. Interestingly, the linear increase in *I_2D_/I_G_* ratios (indicated by blue triangles) up to 40 °C corroborates the formation of thinner graphene flakes [35]. It is worth mentioning that the decreased *I_2D_/I_G_* ratio for the flakes observed in 60 °C sample confirms the agglomeration/re-stacking of small sized sheets (if produced in large quantity), which is consistent with the trend of defect ratios as discussed earlier. This is also linked with FWHM of the G band (given in Fig. 4 (c)) which increases with temperature leading to evolution of edge defects, i.e., progressive formation of graphene sheets. It is to be noted that position of the G band did not shift significantly, which otherwise could be indicative of basal plane defects [36], [37]. The drop of the FWHM-G band at 40 °C might be ascribed to the formation of comparatively large sized graphene sheets in accordance with FWHM-G band ∞ *1/L*; *L* is lateral size of flake [26], [40]. A slight increase of the FWHM-G at 60 °C explains the wide distribution of flake sizes and thicknesses, which might be ascribed to the formation of new graphene sheets of smaller lateral sizes produced as a result of the scissoring effect [42]. Additionally, values of the FWHM-G band stay in its low range of 24–28 cm^−1^, which validates that the defects are most likely due to the edge defects without any significant structural damage [40]. Furthermore, the degree of exfoliation can also be assessed by estimating the FWHM of 2D band [37]. Fig. 4 (c) revealed the decreasing trend of FWHM of 2D band with the least value observed in 40 °C samples, implying the occurrence of thin graphene flakes. The subsequent increase in the FWHM-2D band after 40 °C advocates the formation of stacked sheets as discussed earlier. From the above observations, we interpret that up to 40 °C, GP successfully split apart and exfoliate to thinner flakes with some induced defects mainly confined to the edges.

### 3.3 Morphological analysis

**Fig. 5:**
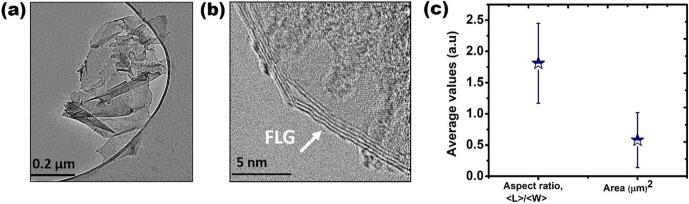
(a) Representative TEM image of graphene flakes exfoliated in 50 %–40 °C samples; (b) High-Resolution (HR-TEM) image of the corresponding flake; (c) average aspect ratio *(<L>/<W>)* and area of exfoliated flakes (with error margins).

In light of the results discussed above, the sample processed with 50 % input power at 40 °C was further assessed by TEM for morphological investigations. Fig. 5 (a, b) display representative TEM image of the obtained graphene flakes and their corresponding high-resolution TEM (HR-TEM) indicating FLG, respectively. Fig. 5 (c) represents statistical information for the aspect ratio *(<L>/<W>*) and area of the exfoliated flakes. From TEM observations, *<L>/<W>* ≠ 1 indicates the formation of asymmetric elongated flakes [43], which is a characteristic feature of ULPE graphene flakes, especially of larger flakes in size. The estimated area of all the registered flakes (∼50) is found to be ∼0.58 ± 0.44 μm^2^ determined with *Image J* software.

### 3.4 Acoustic pressure measurements

**Fig. 6:**
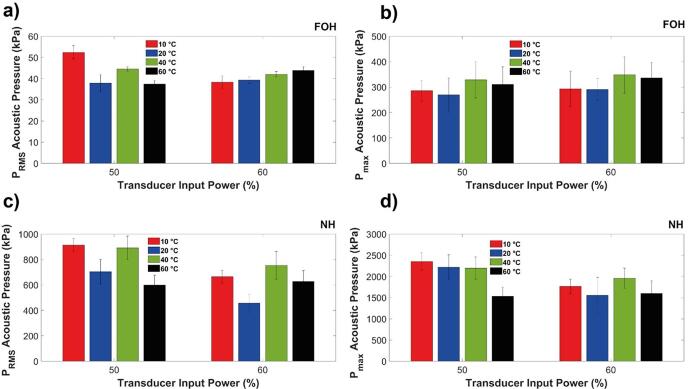
Acoustic pressure measurements taken with sensors for solution temperatures of 10 °C, 20 °C, 40 °C, & 60 °C, comparing 50 % and 60 % transducer input power. FOH measurements of a) RMS pressure; b) P_max_. NH measurements of c) RMS pressure; d) P_max_. Note different Y-axes scales.

Acoustic pressure measurements were taken to complement the characterisation analysis of the graphene samples. Root means square (RMS) pressure measurements with the FOH (Fig. 6(a)) revealed that ULPE at 10 °C for 50 % input power generated the largest pressure, followed by that of 40 °C. An input power of 60 % produced an upward trend with increasing temperature (correlating to decreasing nanosheet thickness in Fig. 2(a), (b)), at 60 °C closely followed by 40 °C, generating the largest pressures. However, even if RMS pressures did not significantly differ between all temperature levels, it is interesting to note that at 40 °C for both input powers (50 % and 60 %) they were almost identical, implying some sort of flexibility in choosing the appropriate power setting. For the maximium recorded pressures (P_max_), Fig. 6(b), it is clear that pressure surges from acoustic waves (mainly from the incident source at 24 kHz and the 2nd and 3rd harmonic, as well as plentiful of sub-harmonics (Fig. 8)) generated the largest pressures at 40 °C. The P_max_ obtained from FOH showed a similar trend with increasing temperature. Since the FOH is calibrated between 300 kHz and 30 MHz (Section 2) it is primed to detect SWs released upon bubble collapse (previously demonstrated as the primary exfoliation mechanism during ULPE [44]).

The P_RMS_ obtained from NH produced the same trend as seen in Fig. 6 (a) with 10 °C and 40 °C giving rise to the highest pressures at 50 % power. This was also the case while using the sonotrode at 60 % input power. Analysis of the P_max_ showed that ULPE at 10 °C and 50 % input power, and 40 °C and 60 % input power generated the highest acoustic pressures (Fig. 6 (d)). The lower calibration range of the NH (8–400 kHz) is primed to detect cavitation activity of lower frequency oscillating bubbles and subharmonics (associated with the periodicity of SWs [45], [46] as well as acoustic pressures from the driving frequency with the corresponding harmonics and ultra-harmonics [47]). In most cases, we see a solution temperature of 40 °C registering the largest acoustic pressure, correlating with the characterisation of the produced graphene samples, indicating that larger pressures facilitate exfoliation. As previously discussed, despite 10 °C solutions generating the largest pressures in some cases (and also demonstrated elsewhere [22]), the dispersion of the bulk graphite is hindered at this low temperature (see Fig. 2(d)), in addition to the increased surface tension in the solution (see Table 2 for physical properties of water), which decreases the efficiency of graphene exfoliation. The pressures recorded at 60 °C (Fig. 6(c)-(d)) may also indicate that the scissoring defects observed during characterization results are a consequence of the solution temperature being deleterious to the graphite, as opposed to the cavitation impact, as much lower acoustic pressures were measured for this temperature.

**Table 2:** Physical properties of the DIW i.e. density [48], viscosity [48], vapor pressure [48] and surface tension [49] at different temperatures.

Temperature (^o^C)	Density (kg/m^3^)	Viscosity (N s/m^2^) × 10^−3^	Vapor Pressure (kPa)	Surface Tension (N/m)
10	0.999	1.307	1.23	74.2
20	0.997	1.002	2.33	72.9
40	0.992	0.653	7.37	69.6
60	0.983	0.467	19.92	66.0

**Fig. 7:**
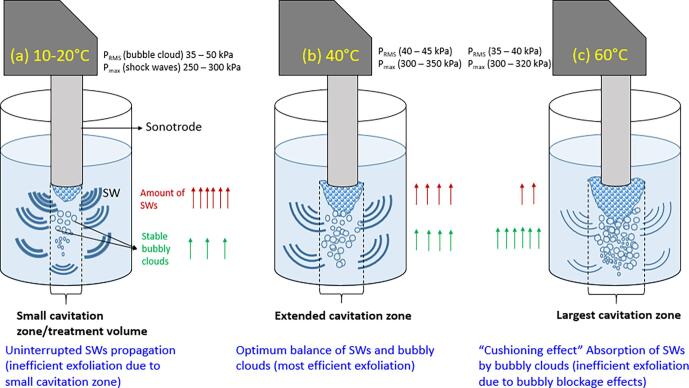
Schematic representation of underlying mechanism for the effect of temperature on graphene exfoliation; (a) 10–20 °C; (b) 40 °C; (c) 60 °C. Note the thickness of SW curves indicating their intensity as captured by the acoustic sensor.

The enhancement in graphene exfoliation with temperature (as discussed previously in section 3.1) can also be explained using the schematic presented in Fig. 7 and in line with our previous work in [22]. The arrows shown in the figure indicate the quantity of SWs and bubbles/bubbly clouds in the bulk liquid. Thickness of SW curves indicates their intensity. At low temperatures (10–20 °C) (Fig. 7(a)), the cavitation zone is largerly restricted under the tip of the sonotrode causing lesser exfoliation of graphite particles, which intrinsically affects the final concentration of the produced graphene. At the same time, smaller bubbly clouds at low temperature regimes allows the undisturbed propagation of SWs (driving mechanism of exfoliation [44]) reaching the acoustic sensor while registering the maximum acoustic pressure as shown in Fig. 6(a) and (b). On the other hand, at high temperature (60 °C) (Fig. 7(c)), despite having the largest cavitation zone, numerous bubbly clouds lead to the absorption of SWs (“cushioning effect” [22]) and reduce their intensity (represented by thinner SW curves), which also results in inefficient exfoliation. Therefore, at 40 °C (or intermediate temperature regimes) (Fig. 7(b)), there is a trade-off between both a larger cavitation zone and substantial SW emissions, which is favorable for efficient exfoliation.

**Fig. 8:**
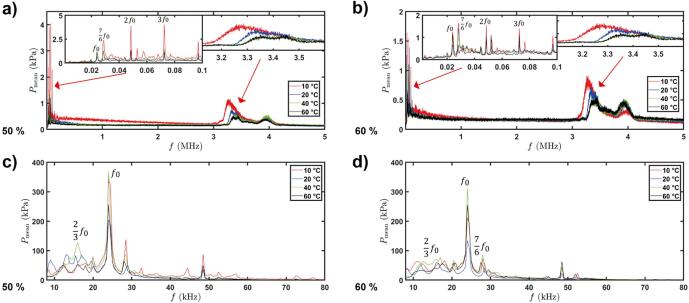
Acoustic pressure spectrum obtained with sensors for solution temperature of 10 °C, 20 °C, 40 °C, & 60 °C, comparing 50 % and 60 % transducer input power using FOH (a & b) and NH (c & d). Note different Y-axes scales.

Therefore, combining the data from both calibrated sensors we can produce a wide frequency range from 8 kHz up to 30 MHz of registered pressures to analyse our cavitation activity occuring during ULPE under various temperature and input power conditions as shown Fig. 8. Acoustic spectra from the FOH (Fig. 8 (a) & (b)) shows an aspect of SW behaviour in the solutions for 50 % and 60 % input power, respectively. The spectrum depicts the pressure peaks in both low frequency (up to 100 kHz, see top left inset in Fig. 8(a) & (b)) and high frequency (between 3 and 4 MHz, see top right inset in Fig. 8 (a) & (b)) regime. The low frequency peaks (including subharmonic, harmonic and ultraharmonics) are mainly ascribed to the periodic emission of SWs from the primary cavitation cloud collapses [46]. While the peaks in the high frequency range correspond to the inherent feature of the travelling SWs as previously discussed in [23]. The peak formed at ∼3.3 MHz is indicative of SW generation from cavitation bubble collapses (reported previously [50]). The trend showed that increasing temperature reduced the intensity of this peak indicating that using higher solution temperatures can lead to weaker cavitation. Larger number of cavitation bubbles are generated at higher temperatures, disrupting the propagation of pressure surges from SWs and therefore the intensity decays faster, as previously observed in [22] and delineated in Fig. 7. On the other hand, these additional bubbles aid the exfoliation process through vigorous oscillations as seen in [44] hence giving rise to larger pressure at low frequencies as seen in Fig. 8 (c) & (d). In addition to the reduced pressure peak intensity, an apparent shift of the SW peak towards higher frequencies was also observed with the increase in the solution temperature (see top right insets in Fig. 8 (a) & (b)) in the acoustic spectra. This shifting of the peak has been previously ascribed to the change in the speed of sound in the medium [23]. Specifically, Khavari et al. [23] characterised the SW behaviour in various liquids such as water, ethanol, glycerol and ethanol–water mixture and demonstrated that irrespective of the large difference in their liquid properties the wavelength of the propagating SWs remained the same and within the range of 420–450 µm. Interestingly, in this study, the liquid properties, and thus the speed of sound, were also affected by the temperature increments. This shift of the pressure peak of the SW towards higher frequencies indicates that the wavelength is kept in a similar range of 430–460 µm (Table 3), confirming the previous findings [23].

**Table 3:** Wavelength of propagating SW in water for solution temperatures of 10 °C, 20 °C, 40 °C and 60 °C obtained from dominant frequency peak in pressure spectrum profile.

Temperature (°C)	Frequency peak 50 % Input Power (MHz)	Frequency peak 60 % Input Power (MHz)	Speed of Sound [51] (m/s)	Wavelength 50 % Input Power (μm)	Wavelength 60 % Input Power (μm)
10	3.27	3.27	1447	442 ± 3.8	442 ± 4.0
20	3.33	3.33	1481	445 ± 2.2	444 ± 3.3
40	3.37	3.36	1526	453 ± 3.9	455 ± 3.4
60	3.42	3.43	1552	453 ± 4.8	453 ± 5.9

Acoustic spectra from the NH (Fig. 8 (c) & (d)) revealed that ULPE at 40 °C produces the largest fundamental frequency harmonic (as well as sub-harmonics at 16 kHz noticeable for both input powers in Fig. 8 (c) and (d) and 28 kHz in Fig. 8 (d)), showing that this temperature contributes to exfoliation through bubble oscillating forces possibly overimposed to the incident wave (thus the peak at 24 kHz is higher than the other temperature levels), but most importantly via periodic SW emissions (source of sub-harmonics [45], [46]). Another interesting observation is that with the further increase of the temperature to 60 °C the pressure peak is significantly suppressed. In particular, the high pressure peak at 24 kHz at 40 °C in Fig. 8 (c) and (d) in conjunction with the highest measured pressures in Fig. 6 (c) is about 45 % and 23 % higher than the corresponding peaks at 60 °C (260 kPa, Fig. 8 (c)) and (255 kPa, Fig. 8 (d)) respectively, indicating the obstruction or absorption of the sonotrode energy from the larger cloud of bubbles (extended bubbly clouds) [22].

Thus, it can be deduced that we can regulate the temperature and input power to such an extent that the intensity of the SWs is diminished (Fig. 8 (a), (b)), i.e. about 50 % input power in the case of 40 °C, by the bubbly surroundings, but at the same time there remains sufficient number of SWs (the reason being the sub-harmonic peak at 16 kHz in Fig. 8 (c), (d) is dominant for 40 °C [46]) that are able to reach and interact with the suspended graphite particles. At the same time, the populated bubbly structure (increasing the temperature increases the tendency of more cloud formations due to higher vapour pressures (Table 2)) vigorously oscillates at the incident frequency and corresponding harmonics, synergistically promoting a gentle exfoliation of graphite, generating high quality graphene flakes. Results are in-line with our previous work, where we showed that a combination of high and low frequencies have the potential to alleviate the powerful SWs and populate the liquid with tiny bubbles that can also expedite the exfoliation process and produce high-quality flakes [13]. Hence, the key for successful and high-quality exfoliation is the control of temperature and power that will induce the right amount of acoustic energy to promote gentle exfoliation. Overall, analysis of the cavitation activity provided evidence that the 40 °C solution would aid the facilitation of gentle exfoliation, and supported the characterisation analysis, which manifested high quality FLG flakes.

## 4 Conclusions

A systematic study of temperature controlled low frequency (24 kHz) ULPE configurations was performed to gauge the degree of exfoliation as a function of temperature with both characterization studies and acoustic pressure measurements. Based on the results, we conclude that ULPE process at 40 °C at the studied input powers (we also showed that slightly higher input power, and as expected, does not affect the quality of exfoliation but offers flexibility to the process) in pure DIW for 2-hours reduces the thickness of graphite crystallites to FLG, with some induced edge defects which are unavoidable in ULPE processes. Results are also in a very good agreement with previous estimations for the best sono-exfoliation conditions based on acoustic pressure measurements [22]. It is demonstrated that the right amount of acoustic energy and related cavitation patterns controlled by the input power and temperature induce gentle but efficient exfoliation of high quality FLG with an area of ∼0.6 μm^2^. The contribution of the SW induced pressure was shown to be a useful factor in monitoring and optimising ULPE. In addition, an upward frequency shift along with reduction in high frequency (∼3 MHz) pressure peak intensity of SWs was observed with the increase in solution temperature. Interestingly, the corresponding wavelength of SWs is independent of the liquid temperature and kept in the range of 430–460 µm for all the solution temperature regimes. Even though the selection of low temperature environments may be desirable to produce a high intensity shock pressure field necessary for promoting ULPE, there is always a trade off that exists between the amount of emitted SWs and the extent of cavitation zone (bubbly cloud) formation required to achieve an optimum balance between the two that maximises the exfoliation output. The ability to use pure water to exfoliate graphene with good structural characteristics and reasonable size will stimulate its exploitation in medical applications such as cell imaging, bio-sensing, tissue engineering, cellular interactions in neuroscience and ecotoxicological studies.

## Data availability

The data that support the findings of this study are available upon request from the corresponding author.

## Declaration of Competing Interest

The authors declare that they have no known competing financial interests or personal relationships that could have appeared to influence the work reported in this paper.

## Data Availability

Data will be made available on request.
